# EHMTI-0113. Headaches associated with symptomatic epilepsy in adults

**DOI:** 10.1186/1129-2377-15-S1-C4

**Published:** 2014-09-18

**Authors:** S Atic, D Atic, R Amanovic Curuvija, M Savic

**Affiliations:** 1 Neurology, Hospital for Cerebrovascular Diseases Sveti Sava, Belgrade, Serbia; 2 General Medicine, Health Center Vozdovac, Belgrade, Serbia

## Introduction

Headache is a symptom very often present in patients with epilepsy.

## Aims

The aim of our study was to find the characteristics of headache in adults patients with symptomatic epilepsy.

## Methods

We studied patients with symptomatic epilepsy and headache using standard protocol:neurological examinations, Color duplex ultrasound, Transcranial ultrasound, Electroencephalography ( EEG), Computerized tomography (CT ) and MR/MRA (angiography ).

## Results

82 patients were observed , 52 female and 30 male, age range 39 to 82.All of them were suffering by acute or chronic headache and symptomatic epilepsy. Preictal headache was present in 8 ( 9,75% ) patients, postictal in 27 ( 32,92% ) and interictal in 47 ( 57,1%) patients. Among the patients with postictal headache 13 ( 48,14% ) had migraine, 9 ( 33,33% ) tension -type of headache and 7 ( 25,72% ) other headaches. Among the patients with interictal headache 28 ( 59,57% ) was migraine, 15 ( 31,91% ) combined migraine and tension-type and 7 ( 14,89 %) other headaches.The majority of seizures were symple focal seizures.

## Conclusions

1)Among the patients with symptomatic epilepsy interictal migraine was commonly than other headaches.

2)The majority of seizures in patients with headache and symptomatic epilepsy were simple focal seizures.

3)The comorbidity may be important in the choice of treatment of headache.

